# Feasibility of a Website and a Hospital-Based Online Portal for Young Adults With Juvenile Idiopathic Arthritis: Views and Experiences of Patients

**DOI:** 10.2196/resprot.3952

**Published:** 2015-08-14

**Authors:** Judy JW Ammerlaan, Lieske W Scholtus, Constance HC Drossaert, Harmieke van Os-Medendorp, Berent Prakken, Aike A Kruize, Johannes JW Bijlsma

**Affiliations:** ^1^ University Medical Center Utrecht Department Rheumatology and Clinical Immunology Utrecht Netherlands; ^2^ University of Twente Department Psychology, Health and Technology Enschede Netherlands; ^3^ University Medical Center Department Dermatology and Allergology Utrecht Netherlands; ^4^ University Medical Center Utrecht Department Pediatric Rheumatology and Immunology Utrecht Netherlands

**Keywords:** eHealth applications, feasibility, website, digital portal, young adults, juvenile idiopathic arthritis

## Abstract

**Background:**

To improve knowledge and to encourage active involvement of young adults with juvenile idiopathic arthritis (JIA), an informative website with written and video information and an online portal with access to the personal medical record, self-monitoring, and e-consult functionalities were developed. Before implementing these applications in daily practice, it is important to gain insight into their feasibility in terms of ease of use, perceived usefulness and intention to use.

**Objective:**

The aim of this study was to evaluate and to examine the feasibility of the website and the online portal for young adults with JIA.

**Methods:**

A qualitative, feasibility study was conducted among the first users: 13 young adults with JIA. After provided access to the website and online portal, patients were interviewed on perceived usefulness, ease of use, and intention to (re)use the applications.

**Results:**

Participants in the study considered the website and online portal as useful and easy-to-use. New medical information and feedback would motivate them to revisit the applications again. On the website, videos showing other young adults, telling how they handle their condition, were found as the most useful. On the portal, access to their medical records was most appreciated: it made the young JIA patients feel in control and it helped them monitor symptoms and disease activity. e-consults were thought to facilitate communication with physicians.

**Conclusions:**

The young adults considered both the website and the online portal as feasible, but they also had valuable suggestions to improve accessibility and use. Based on these findings, a news and event section was added on the website and a direct link was made to a discussion board and social media. To provide and support health information, the website is actively used in daily care. Considering the online portal, the use of self-monitoring tools and e-consult can be stimulated if there is direct linkage to treatment and feedback from the multidisciplinary team. Feasibility testing, before implementing the website and online portal in daily practice, has proven to be a valuable step. Results led to improvements in terms of integration into standard care and topics for further research.

## Introduction

Living with a chronic rheumatic disease is challenging at any age. However, these challenges may be particularly difficult for young adults with juvenile idiopathic arthritis (JIA), since their chronic condition and treatment affect both physical and socio-emotional development [1,2]. One of the main challenges young adults with JIA have to deal with is to take over from their parents the responsibility for their own illness and treatment: they have to become a self-manager [3,4].

In general it is believed that eHealth might contribute to self-management, especially for young adults [5-7]. For this study, we followed the definition of eHealth by Eng: “The use of emerging information and communication technology, especially the Internet, to improve or enable health and health care” [8]. The use of health information websites and eHealth applications, including online portals in disease management, disease prevention, and health promotion is well-reported [9,10]. Unfortunately, many eHealth projects fail to survive the pilot phase and studies that focus on the effectiveness of eHealth applications often do not show any long term effects [9-11]. Also, much is developed but not everything is used [10,12,13]. For the actual use and acceptance, evaluation and testing of the feasibility before implementing the techniques in daily practice is crucial [11,14-16]. Several frameworks have been introduced to increase the uptake and to examine the feasibility of eHealth applications. Among them, the technology acceptance model (TAM) [15,17] stands for a prediction and explanation model of the end-users reaction to a technological innovation. The model states that use or acceptance of a particular innovation can best be predicted by an individual’s intention to (re) use the innovation. A comparative model, the Holistic Framework of Gemert [11] suggests that developers of eHealth applications should be aware of interactions between technology, people, and their social-cultural environment. Involvement of end-users in developing eHealth applications is considered to be one of the crucial aspects of acceptance of the tools themselves.

In our specialized transition outpatient department, young adults with JIA and their parents receive multidisciplinary care from a pediatrician and a clinical nurse specialist, in order to support the process of acquiring self-management skills and to guarantee a well-coordinated, continuous process of health care between child and adult [5]. To improve knowledge of the disease and to encourage active involvement in this transition process, we developed an informative website and a hospital-based online portal. The website contains information about medical issues and how to deal with consequences of having a rheumatic disease, such as feeling blue, exercise, work, study, relationships and intimacy.

With our secured online portal the young adult has direct access to his medical record; he is enabled to send an e-consult and may use self-monitoring tools including activity diaries and pain questionnaires. These applications were developed in close cooperation with patients to fit the applications to the specific needs and preferences of this group. Therefore, young adults of the Dutch Youth Network of Rheumatology were asked to perform a central role. In interactive workshops, organized by the multidisciplinary team of the transition outpatient clinic, they determined, together with the professionals, the content and structure of the website and portal.

Both applications may be promising to reach young adults with arthritis and to stimulate their self- management behavior, given their access to and high rates of use of the Internet [7,18,19]. Before implementing these applications in daily practice, it is important to gain insight into their feasibility. Therefore, the aim of this study is to evaluate and to examine the feasibility of the website and the online portal for young adults with JIA.

## Methods

### Design

A qualitative feasibility study with semistructured, (audiotaped) interviews was conducted among the first users of both eHealth applications in order to explore the views and experiences of the young adult JIA patients with regard to the feasibility outcomes: ease of use, perceived usefulness, and intention to use. These outcomes are part of the technology acceptance model [17]. The model states that use or reuse of a particular technical innovation can best be predicted by an individual’s intention to (re)use the innovation. This intention is determined by two components: perceived ease of use and perceived usefulness. Perceived ease of use means “the degree of ease, associated with the use of the applications”. Perceived usefulness can be defined as the degree in which a person believes that using the technical innovation would enhance his or her personal situation.

Participants were asked to use the applications for three months “as needed”, without specific instructions, and were interviewed just before or after their subsequent visit to the clinic.

### Study Population

Patients diagnosed with JIA, aged between 16 and 25 years old, being able to read and write in Dutch, with access to a home-based computer with Internet were included in the study. Young adults who already participated in the development of the website and/or portal were excluded.

Recruitment took place at the transition outpatient department of the University Medical Center Utrecht, the Netherlands. All patients who visited the transition outpatient department within a period of three months were asked by their pediatrician or rheumatologist to participate in the study. An information letter and informed consent was handed out which they could return by post.

A convenience sample was used: all patients who returned the informed consent within two weeks after their visit were included in this study. Because it was a feasibility study with a qualitative design, we aimed to include at least 12 participants. According to the ethics guidelines of our hospital, the nonexperimental and noninvasive nature of this study made ethical approval unnecessary.

### Description of the eHealth Applications

The website and online portal are designed to improve knowledge, self-management skills, and involvement in treatment and care. The website (in Dutch) [20] is publicly accessible and contains information and tips on five themes: (1) treatment and medication; (2) physical exercise, holidays, alcohol, and drugs; (3) relations, sexuality, and pregnancy; (4) dealing with pain, fatigue, and emotions; and (5) study and work. In addition, videos and written stories from other young adults talking about their lives with JIA are presented.

**Figure 1:**
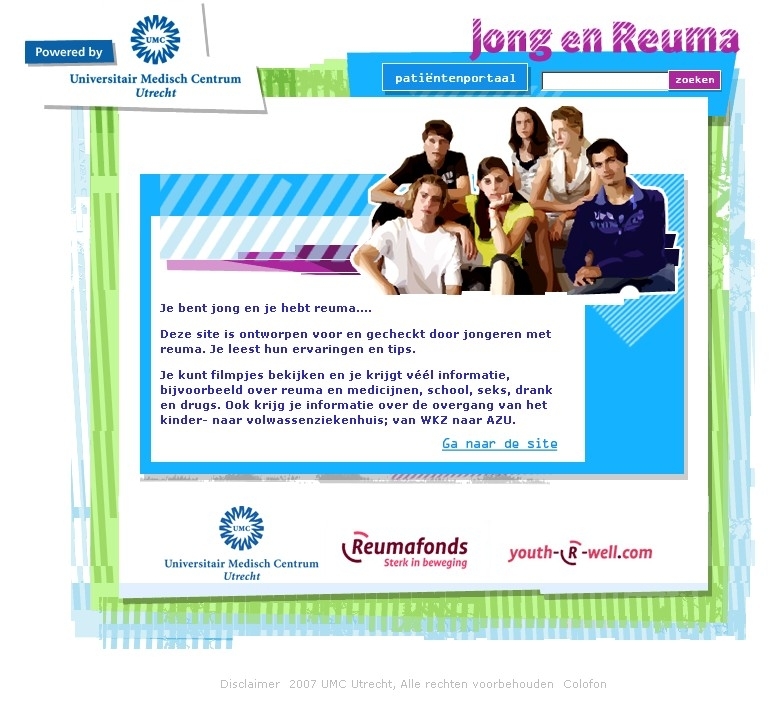
Homepage of the Dutch website.

For an impression of home page of the Dutch website, see Figure 1.

The portal (in Dutch) [21] is only accessible to JIA patients of the University Medical Center Utrecht with a personal log-in code. By using the portal, patients may have access to the following tools: (1) e-consult, through which patients communicate with a clinical nurse specialist; (2) their own medical record, including all written reports of physicians, laboratory results, present medications and appointments with the outpatient clinic; and (3) online self-monitoring, in which patients can fill out self-tests on pain and activities and diaries to monitor their disease.

**Figure 2:**
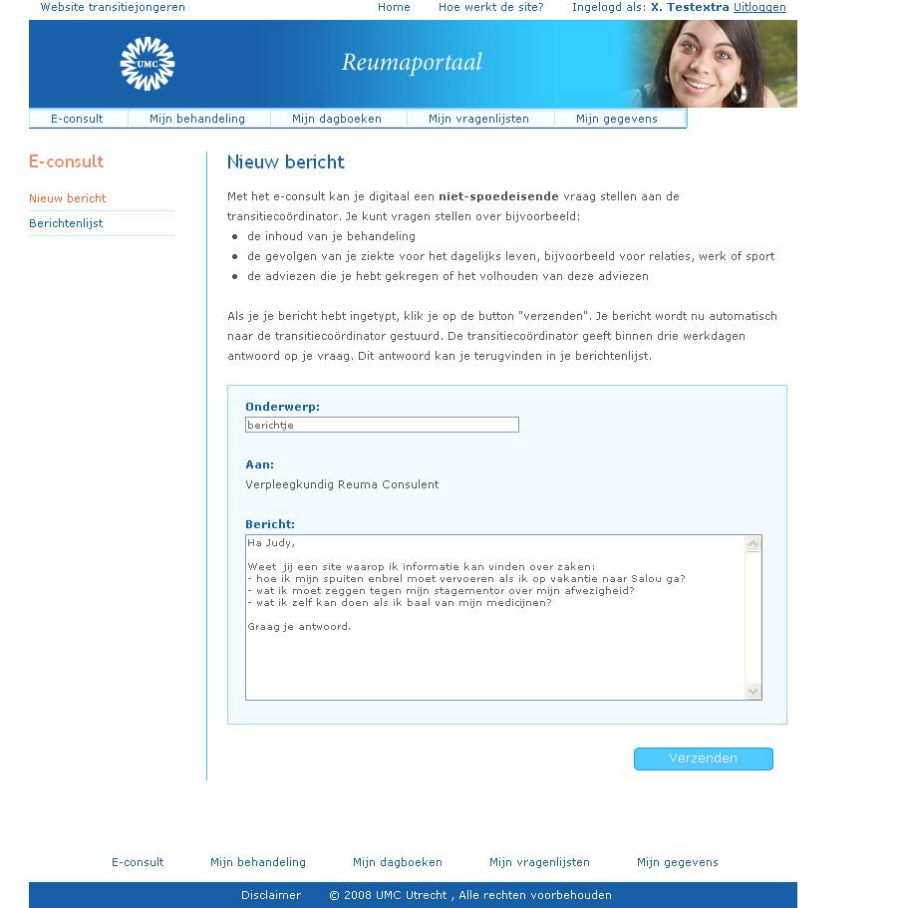
Portal and e-consult tool.

For additional general disease information patients are referred to the website [20].For an impression of the portal and the e-consult tool, see Figure 2.

The content of website and portal are outlined in table 1.

**Table 1:** Content of website and portal.

Website, portal		Content
**Website**		
	Transition	What is transition?
		Practical information about the departments, multidisciplinary team
	About JIA	What is JIA?
		Treatment
	Leisure	Exercise and sports
		Alcohol and drugs
		Holidays/vacation
	Love, sex, and kids	Parents and friends
		Sex and intimacy
		Having kids
		Contraception, pregnancy, and heredity
	Feeling blue	Having a rheumatic disease
		Feeling tired or having pain
		Relaxation
		Feeling depressed
		Adherence and treatment advices
	School, work, and money	School
		Work
		Social insurance
**Portal**		
	Medical record	Appointments
		Laboratory results
		Written reports of physicians
		Actual medication
	e-Consult	Sending and receiving e-consults
	Online self-monitoring	Medication diary
		Diary on fatigue
		Pain score
		Activities

### Data Collection

First, participants were asked to complete a brief questionnaire on demographics, illness characteristics, and their general Internet use. Subsequently, a semistructured interview was conducted at the transition outpatient department in a separate, quiet room, by a young independent interviewer (LWS), who was not involved in the care of the young adult.

The first part of the interview was directed at the informative website; in the second part patients’ opinions and experiences about the portal were assessed. For both applications, open-ended questions, deriving from TAM were used to get insight into ease of use, perceived usefulness, and intention to (re)use (see Table 2 for an overview of questions). The interviewer encouraged participants to elaborate on all issues, using probes such as: “why?”, “please, explain…”, “Can you give an example?”, “Can you think of any other …” At the end, the participant was asked to grade the applications with a number, where “zero” stands for “not useful” and “ten” stands for “the most useful”.

**Table 2:** Interview questions on feasibility of the website and portal.

	Interview questions
Use	Have you visited the site/portal?
	Did you visit the site/portal with a specific reason?
	Which parts did you visit?
Perceived ease of use	Did you experience any difficulties in use of the site/portal?
Perceived usefulness	How useful is the site/ portal for you?
	Which parts are most/ least useful?
	Can you describe any benefits, or drawbacks?
	What are the most and least appealing parts?
	Do you miss anything?
Intention to use	Would you visit the site/portal again?
	Would you recommend the site/portal to others?
Suggestions	Do you have any suggestions for improvement?

All interviews were audiotaped. Field notes were made immediately after the interview to record the interviewer’s impression of the responses to the questions and comfort level of the participants with the interview process. An interview lasted between 30 and 45 minutes.

### Data Analysis

All interviews were transcribed verbatim. A generic qualitative approach was used for data analysis, including coding, constant comparison, and categorizing. Data collection and analysis were handled as an iterative process [22]. The first participant was interviewed with open-ended questions on feasibility and suggestions for improvement of the website and portal. In the following interview, the feasibility was asked again and the suggestions made by the former participant were checked. Then the participant was asked to add his own suggestions. The interviewer checked every interview if new themes emerged and asked further about these themes until saturation was achieved and no new information was obtained. The interviewer (LWS) and a member of the research team (JWA) reviewed and coded all transcripts independently. Relevant fragments were first categorized into the main concepts of TAM and were further categorized into subthemes, using inductive analysis. Results were discussed on several occasions and differences were discussed until consensus was reached.

## Results

### Participants

Thirty-eight eligible patients received an information letter and informed consent. Patients (n=19) who returned a signed informed consent were included in the study. No information is available from the other 19 patients who did not respond to the invitation to participate in the study. Six patients dropped out after giving their informed consent, with reasons of exacerbation of illness, vacation, and too busy with school. Finally, 13 participants received the URL address of the website and a log-in code for the portal.

The mean age of the sample was 20 years (range: 17-22 years) and consisted of 12 women and 1 man. Most of the participants (n=12) were being treated by a rheumatologist. The mean duration of illness was 8years (range: 2-20 years). Of the 13 participants, 11 were in high school, and two participants were gainfully employed. They all used the Internet on a daily basis, most participants (n=10) with an average of over two hours a day.

### Informative Website

Of the 13 patients, 12 indicated having visited the website several times. One participant visited the website only once. During their visits, all participants had read at least a part of the written information. Most (n=11) had seen one or more videos and read written life stories. The primary reason for (re)visiting the website was curiosity. Other reasons included searching for specific information or for experiences of other patients.

### Perceived Ease of Use

Participants did not experience barriers visiting the website. Two participants had issues with loading a video, in retrospect due to their own computer and Internet connection.

### Perceived Usefulness

Participants appreciated the website and graded it with a 7.6 (min 6.5, max 9.5) on a scale from 1 to 10. They found the design of the website appealing and the information practical, clear, easy to read, and well-targeted to young adults.

This really is for younger people. I sometimes look at the SLE site but that is mostly for people who are 40, 50 and 60 years of age and their problems. That is not really my cup of tea. This really reveals itself to be more for younger ones.

Female, 21 years

Of all the elements, the videos and life stories were thought to be the best. They enabled recognition and showed new ways to deal with solving problems related to the condition. Patients experienced support and recognition in these stories.

I sometimes think that I am the only one with arthritis. There aren’t many with the same problems. Other people don’t understand this; they don’t see anything on the outside. It is nice to hear people of the same age talking about this problem. That is what I have too! You won’t have to say it yourself; somebody else says it for you and I feel the same things.

Female, 21 years

It really helped me, especially how to cope with fatigue and pain and how to solve it.

Female, 20 years

You can hear and read the experiences from others, things they had to cope with, how they dealt with them. Often you can learn from their experiences, because you recognize them.

Female, 20 years

All five themes (in Table 1) on the website were appreciated positively. The participants thought nonmedical themes such as dealing with pain, fatigue, and emotions, physical exercise, holidays, alcohol and drugs, sexuality, study and work, were most appealing.

Some young adults missed detailed information on new developments in medication. Others missed a forum. They thought a link to an existing Dutch discussion board for young adults with JIA and to detailed information about medication on other reliable websites would be valuable supplements.

All participants, except for the youngest, indicated that no or only little new information was added to their knowledge and skills by their visit to the website. Nevertheless, the website was considered useful as a confirmation of what they already knew.

I read that it is important to structure your medication. You have to learn that this is important. Also that before visiting a doctor it’s important to make a list with questions. All things you know but important to read again.

Female, 21 years

I think I am beyond that age. I was 15 when I got arthritis. I think I would have had more benefit from this between my 15^th^ and 18^th^. I’m beyond that now. I know what it is; I know how it influences my lifestyle, my alcohol use during the holidays. So I recognize all the subjects but I already have my own opinion about these. It doesn’t add anything for me.

Female, 22 years

The youngest participant felt she found new and relevant information on the website.

In the hospital, they always talked about JIA. I asked myself: what does that mean? I felt really stupid. I didn’t dare to ask. I read on the website that it was Juvenile Arthritis, my disease.

Female, 17 years

Finally, participants indicated that the website might be useful for questions of their relatives:

It is very convenient for yourself and your surroundings. My friend sometimes wants to know more and for him it is also a good site to refer to. It is clearly explained what the disease is and how to cope with it.

Female, 21 years

In summary, the website was considered useful for three reasons: (1) to find or re-read information (mostly already known), (2) to help in explaining the disease and its consequences to others, but most importantly, (3) to find recognition, and to see that other young people struggle with similar problems.

### Intention to Use

Half of the participants intended to revisit the website; the other participants however indicated that they would only revisit the website if new information were added, if news was added or if their personal situation changed. They all recommended the website to other young people with a rheumatic disease.

### Online Portal

All participants (n=13) used the portal for a period of two to three months.

Within this relatively short time, access to medical records was used most often, whilst fewer participants used the tools for e-consult (n=4) and four other participants used self-monitoring (n=4).

Most of the participants logged in just after a visit to the hospital. “Curiosity about what the doctor or nurse had written” was the main reason to check their medical record. One participant logged in just before her visit. She wanted to prepare herself and read what was discussed during her last consultation.

### Perceived Ease of Use

The only problem participants reported (n=5) was the log-in code being too long and complex to be remembered, whereas the code could not be changed. Consequently, they used the portal less often than they wanted.

### Perceived Usefulness

Participants graded the portal with a mean grade of 7.8 (min 7.5, max 8.0) on a scale from 1 to 10, and mentioned several advantages (see Table 3).

**Table 3:** Usefulness of the different parts of the portal, as mentioned by the participants.

Mentioned effects, advantages		Example quote
**Access to medical record**		
	Feeling more in control	“*It’s very functional to be able to check your appointment, to check the blood values and what was said during a consult. Being able to control your own treatment.” [Female, 22 years]*
	Keeping pace with illness activity	“*You have the most recent values and you are able to compare them with the latest values. I once had a liver condition and could see that on the values, it was obvious.” [Female, 20 years]*
	Reminder tool	“*Recently I wondered what we agree on as treatment and medication and now I was able to look it up. Also when I had an appointment I found out that the appointment was half an hour earlier than I thought.” [Female, 20 years]*
	Being able to share data with others (parents)	“*My parents never accompany me and now I am able to show them the summary of the consult.” [Female, 21 years]*
**e-Consult**		
	Easier communication with the hospital	“*To send a notice is easier than phoning to the hospital. I send a message and it doesn’t matter if it’s inconvenient. If I call, it might be inconvenient.” [Female, 22 years]*
	Better communication with doctor/nurse	“*You’re able to think things through and to explain it better.” [Female, 18 years]*
**Self-monitoring**		
	Facilitates communication with physician	“*If things become worse suddenly, it is handy because you know when it went wrong. The doctor is also able to see when it went wrong. Because sometimes I’m ill and when he asks I don’t know when I was ill.” [Female, 19 years]*
	Provides insight into (the course of) the disease	“*Yes, especially when I thought I’m in pain and very tired, it immediately asks what have you done to prevent it and every time you’re not able to answer this question you know that you should have been less active.” [Female, 20 years]*

Four participants used e-consult, and they considered e-consult helpful because of easier and better communication with the hospital. It enabled the participants to think more about the questions and to explain it better.

Most participants could not think of any disadvantages of the portal; although one participant suggested other people might think access to medical data is not safe. One participant indicated that too much information about her health had sometimes overwhelmed her:

The first time I thought I wish I hadn’t read it. That also applies to the website. However it is good to read things about it. It is about you and therefore scary. On the website it is about different people. The portal is very personal.

Female, 17 years

Participants mentioned various aspects of usefulness which are summarized in Table 3.

The access to their medical records was considered the best feature of the portal, because it enabled participants to check their appointment, to see all laboratory results, to re-read treatment plans, feeling more in control of their own treatment, but also keeping track of the progression of the illness was expressed. The portal also facilitated sharing these data with their parents.

The opinions on the usefulness of the self-monitoring tools were more diverse. Some participants indicated them as useful to discover why they experience more symptoms at certain times. Others stated that it provides insight into the course of their problems like having pain or being tired.

A few young adults stated that they did not like filling in diaries or monitoring tools and did not want to be too occupied with their condition.

### Intention to Use

All participants intended to use the portal again, especially for access to their medical records and using e-consult. Some participants were certain they would not use the diaries and monitoring tools; other participants would use these in case of exacerbation of their JIA. Some thought they would use the tools only if their physician asked for it, or their physician would use the information during the consultations.

Participants suggested the portal might be even more attractive if elements were added, including a facility for online appointments, access to x-rays, printing forms for blood collection before the consult, and an overview of physicians and clinical nurse specialists and their consulting hours.

## Discussion

### Principal Findings

In this study, the first users of an informative website and an online portal with opportunities for e-consult, access to medical records, and tools for self-monitoring were asked to evaluate the feasibility of these applications on ease of use, perceived usefulness and intention to (re)use. Both eHealth applications were found easy-to-use, and the young adults considered them as “clear and understandable” and useful.

### Informative Website

On the website, the videos were considered as visually appealing and interactive and as a more pleasant way to learn compared to written information. This appreciation of videos as a source of information was also shown in similar studies in patients with JIA of the same age [6,14]. After seeing other young adults with JIA talking about their lives, some participants felt able to deal/cope with their own condition more adequately. This might indicate effects of modeling and persuasive information, which in fact are methods for enhancing self-efficacy and self-management behavior [23,24]. Most participants indicated they did not encounter information on the website which was new to them, which might be explained because they were relatively experienced patients, as indicated by mean disease duration. The website might be especially helpful for the relatively inexperienced patients, which would be a valid reason for developing these tools preferably for younger patients and for patients recently diagnosed with JIA. These results are in line with data concluding that patients who feel insecure, concerned or inexperienced are more in need of health related information [25,26]. Otherwise, participants from this study indicated that changes in their personal situation and new information might encourage the patient to visit the website again. Based on these results, the website is now actively used at the transition outpatient department as the main source to provide and support health information. The young adult is stimulated by all members of the team to use the site for adequate, additional information. Also, a section with news and events is added to the website in order to stimulate re-visiting the website. In these sections, new information on “being young and having a rheumatic disease” is regularly posted. For this purpose, we created links to the website of the Dutch Youth Network on Rheumatology [27] and the Dutch Arthritis Foundation.

### Online Portal

Participants indicated that access to their medical record was the most useful tool of the portal, increasing their feelings of being in control and helping them to monitor their symptoms and disease activity. Similar results are also found in a large study on access and usage of Web-based communication among adult patients with a chronic disease [28,29]. In our study, young adults “felt more in control” because they could check their appointments, blood values, and “what was said during a consult”. Feeling more in control as part of perceived usefulness in relation to access to a patient portal was also positively rated and recognized by adults with type 2 diabetes [30], and adults with rheumatoid arthritis [31].

Participants in our study expressed the thought that e-consult may lead to easier and better communication with physicians. This result is also reported in other studies on use and acceptance of electronic communication among patients with cancer [32], where email or e-consult were preferred over telephone contact. The finding that most of our participants did not use the e-consult or self-monitoring tools might be attributed to the short period of time between receiving the log-in code and the interview (two to three months). Our finding that participants were only moderately enthusiastic about self-monitoring tools is in concordance with qualitative data on the development of a health information technology mode: the uptake of self-monitoring tools and also e-consult is stimulated if both have a linkage to treatment and to feedback from physicians [25,33]. Consequently, in the implementation phase, the multidisciplinary team will stimulate the young adult to use the tools. Also, the team will address active responses.

### Limitations of the Study

Limitations for this study include participation of only a small group of first users of the applications. Although the sample seems to be representative as to age and illness duration [34], given its small size along with the aim of this study, generalization to the whole population of young adults with JIA is limited. Because it was a convenience sample, no effort was made to recruit the same amount of men and women, which resulted in a high percentage of women in this study. Although JIA is more prevalent in women [2], because of the small groups we cannot make a useful comparison. More research on the results within another group of young adults with JIA or another chronic disease is needed. The participants had a limited amount of time to use the applications; so only their first experiences with the website and portal were collected. No information is available on the patients who did not respond to the invitation to participate in this study. Data collection was performed by rather time consuming semistructured interviews. Within the context of this study, we chose this method to meet the young adults, ask their opinions, and to have the opportunity to encourage them to elaborate on issues of feasibility. In case of a larger sample size, other methods including an online focus group might have been suitable. Focus groups also used to discover perceptions of the participants, often on a limited number of issues, may facilitate the interaction between participants. A recent study on testing feasibility of an eHealth intervention for binge drinking among young people used an online focus group to explore acceptability among 110 adolescents and young adults [34]. For practical reasons, to avoid extra travel time or time lost at school, we chose to plan the interviews before or after a consult. Because this choice might influence our results, the interviews were set up in a separate room, by a young independent interviewer who was not involved in care. Because of their duration of disease, participants were well-accustomed to usual care in our hospital.

### Feasibility Testing

With this study we highlighted the importance of conducting feasibility testing prior to implementation of eHealth applications in daily practice. In information science, the involvement of the end-user in the development process of eHealth applications is considered to be a crucial factor for the actual uptake of the applications in daily life [35,36]. A review of social media in adolescent and young adult health care underpinned our results that targeting health information, based on the needs of the specific group, could stimulate the actual use of an eHealth tool [37].

In this study, we focused on feasibility testing but in the actual development of website and online portal, we collaborated actively with young adults as well. They decided to a large extent the content, layout and structure of both applications, based on their needs and preferences. The positive outcomes of our study may be attributed to this earlier collaboration. The TAM model, used in this study [17], has already evolved towards a Health Information Technology Acceptance Model (HITAM) [25] adding (behavioral) factors such as HIT self-efficacy and health beliefs/concerns.

Based on the results of this study and the high use and acceptance within the group of young adults, we can conclude that the Internet can be a promising tool to provide health information and improve self-management among young adults with rheumatic diseases. This point is also recognized in other studies of young adults with a chronic disease like HIV [38] or Spina Bifida [39] on use and preferences regarding eHealth. Also Stinson’s study [14] of young adults with JIA showed similar outcomes. Young adults with JIA believe that “*Web-based interventions are a promising avenue to improve the accessibility and availability of JIA management strategies”* [14].

Also mentioned in the HITAM is the “subjective norm”, indicating that when a HIT (eg, website or portal) is imbedded in social networks, consumers are more likely to have a positive attitude towards acceptance. Based on these assumptions and the results of our study, we linked the website to a discussion board and social media. However, here some concerns have to be expressed. Several studies show that most young adults primarily use the Internet and social media for contact with peers, for home work or for leisure activities [37,39-41]. Little is known about the actual use of these media in relation to this group and health care. Future research is needed to gain insight into the use and acceptance of these media in relation to health.

Further, website and portals are increasingly used in health care, most in addition to usual care. Further research into the consequences of replacing parts of usual face-to-face care by eHealth interventions, including cost-effectiveness, will be needed.

### Conclusion

The young adults appreciated both website and online portal as feasible but they also had valuable suggestions to improve accessibility and use. Based on these findings, a news and event section was added on the website and a direct link was made to a discussion board and social media. To provide and support health information, the website is actively used in daily care. As concerns the online portal, the use of self-monitoring tools and e-consult can be stimulated if there is a direct linkage to treatment and feedback from the multidisciplinary team. Feasibility testing, before implementing the website and online portal in daily practice, has proven to be valuable. Results led to improvements in terms of integration in usual care and topics for further research.

## Abbreviations

JIA: juvenile idiopathic arthritis
HITAM: health information technology acceptance model
TAM: technology acceptance model
